# How does trust affect acceptance of a nuclear power plant (NPP): A survey among people living with Qinshan NPP in China

**DOI:** 10.1371/journal.pone.0187941

**Published:** 2017-11-27

**Authors:** Qunying Xiao, Huijun Liu, Marcus W. Feldman

**Affiliations:** 1 Department of Management Engineering, Engineering University of CAPF, Xi’an, China; 2 School of Public Policy and Administration, Xi’an Jiaotong University, Xi’an, China; 3 Morrison Institute for Population and Resource Studies, Stanford University, Stanford, California, United States of America; University of Liverpool, UNITED KINGDOM

## Abstract

It is difficult to know whether different dimensions of trust have different effects on local residents’ acceptance of nuclear power plants (NPPs). In previous research such trust has been considered as a single dimensional concept. This paper divides trust into goodwill trust and competence trust, and we explore the ways in which trust affects acceptance of NPPs through structural equation modeling. A survey of 491 people was conducted in Haiyan County, China, where the Qinshan nuclear power plant is located. We find that goodwill trust is significantly correlated with competence trust, and each can indirectly promote residents’ acceptance of NPPs but by different paths. Goodwill trust improves acceptance of NPPs by decreasing risk perception, while competence trust improves acceptance of NPPs by increasing benefit perception. However, the associations between goodwill trust and benefit perception, competence trust and risk perception, are not significant.

## Introduction

After the Fukushima nuclear accident in Japan in 2011, the major nuclear power countries were in a dilemma. The government of Japan shut down most of its NPPs. Global nuclear power supply decreased from 2629.82 TW.h in 2010 to 2476.22 TW.h in 2016 [1]. The accident changed people’s attitude to NPPs so much that the governments of Germany, Italy, Belgium, Switzerland, and Sweden announced plans for withdrawing nuclear power, and anti-nuclear movements emerged all over the world. However, the government of China acted against this trend, implementing an ambitious nuclear power strategy [2]. According to the data from International Atomic Energy Agency (IAEA) updated in May 2016, Chinese nuclear reactors in construction and planned account for 33.9% and 36.8% of the global percentages, respectively [3], which is the highest growth rate in the world. There were only 5 NPPs with 16 nuclear reactors commercially operated in mainland China in 2011, accounted for 3.25% of global nuclear generation, while the number of NPPS and reactors ran up to 12 and 38 in 2017, accounted for 8.52% of global nuclear generation [4–6]. The country is developing nuclear power in the fast lane. Does this mean that Chinese society has more acceptance and trust in nuclear power technology and NPPs?

Though there was a great evolution in the attitudes to nuclear power, worldwide lack of trust in NPPs has resulted in low acceptance of NPPs. Globescan investigated 19 countries in 2005, and revealed that there was still worldwide distrust of nuclear power technology, though the percentages of people in China (not living around NPP sites) trusting in nuclear power (38.7%) were indeed significantly higher than in other countries (29.8% in average) [7]. However, as what happed in most western societies, the attitudes to NPP are diverse. Some people support the development of NPP while some others are against it. Actually, some anti-nuclear protests have emerged in China in the past, for example the protest activities in Hongkong 1986 (aganinst Dayawan NPP), in Fushan of Shandong Province 2006 (against Hongshiding NPP), in Penzhe county of Jiangxi Province 2012 (against Maozhishan NPP), in Jiangmen of Guangdong Province 2013(against Longwan Nuclear Feaul Project), in Lianyungang of Jiangsu Province 2016(against Lianyungang NPP).

Trust is a positive expectation about the function of others in potentially risky circumstances [8], which has been proposed as necessary conditions for formation of alliances [9].The diversity in acceptance of NPPs and trust in nuclear power has been explained by social and cultural difference in previous studies. However, a nuclear power plant (NPP) is a typical adjacency avoidance project. People dislike having the location of an NPP within a certain distance from where they work or live. Higher acceptance of NPPs and more trust in nuclear power doesn't guarantee lower opposition to NPPs construction. Most people distrust not only nuclear energy technology itself but the whole nuclear power industry. In addition, scientists who support the development of nuclear energy, and governments who assert that nuclear power is safe are also not trusted [10]. It seems that trust problem of local residents should be considered seriously during the rapid development of nuclear power in China. To resolve the problem the influencing mechanisms of trust on acceptance of NPPs must be explored precisely.

Previous research has shown that trust might directly influence risk or benefit perception; thus increasing trust can help to increase the acceptance of NPPs [11–14]. The social and culture difference that acceptance of NPPs risk may be correlate with age, gender, education and income have also been broadly explored in previous studies. However, in these studies trust was measured using a single dimension, rather than a set of diverse dimensions. Blomqvist reviewed studies of trust in social psychology, philosophy, economics, and law (contract science), and found that trust encompasses credibility, sincerity, hope, and belief in the competence of the partner [15], which suggests that trust entails not only emotional acceptance but also affirmation of the competence of the partner. Obviously, to promote NPP acceptance it is necessary to known the influencing paths of trust components. Yet it is still unknown especially among the residents living with NPPs. Actually, the main diverse in attitudes to NPPs may be explained by the effect of “adjacency avoidance projects” which suggests that the residents nearby the sites of NPPs are more likely to be against the project rather than social status. Thus this paper will focus on the trust mechanism of residents living with NPPs by sampling the respondents living or working within a 30 kilometers radius of the NPPs and explore different aspects of trust that might underlie the acceptance of NPPs. Given the worldwide lower distrust on NPPs and the phenomenon of “adjacency avoidance projects” is universal, we try to built a model according to previous theoretical and empirical studies in different countries generally and tested the model in a specific Chinese context.

### The concept dimensions of trust

A large number of studies divide trust into ‘cognitive trust’ and ‘affective trust’ (e.g. [16–19]). Cognitive trust emphasizes that an individual looks for rational reasons to believe others, for example, by analyzing whether the partner´s interest is consistent with that individual’s own [20,21], and whether the partner´s resources and capacity are sufficient to manage risk [22]. Affective trust, on the other hand, emphasizes that an individual looks for reasons to believe others based on mutual emotional investment [16], for example, by determining whether the partner is sincere and reliable [15,23] or whether the partner is trustworthy and hopeful [17].

But affective and cognitive trust are relatively broad descriptions. To make the boundary between them clearer, trust is also divided into ‘goodwill trust’ and ‘competence trust’, where goodwill trust corresponds to affective trust, and competence trust corresponds to cognitive trust. Goodwill trust refers to whether the partner will make an open-ended commitment to take mutually beneficial actions in risky situations; competence trust focuses on whether the partner is capable of doing what he promises to do [19]. Goodwill trust reflects recognition of reliability and sincerity [16–18,22,24], and predictability of the commitment of the partner [8,19,25]. Competence trust can be interpreted in different ways, such as ability trust [26], technical trust [27], expertise trust [28], tool trust [29,30] or confidence[31], indicating the expectation that others will carry out their roles competently.

### Trust of NPPs

**Fig 1:**
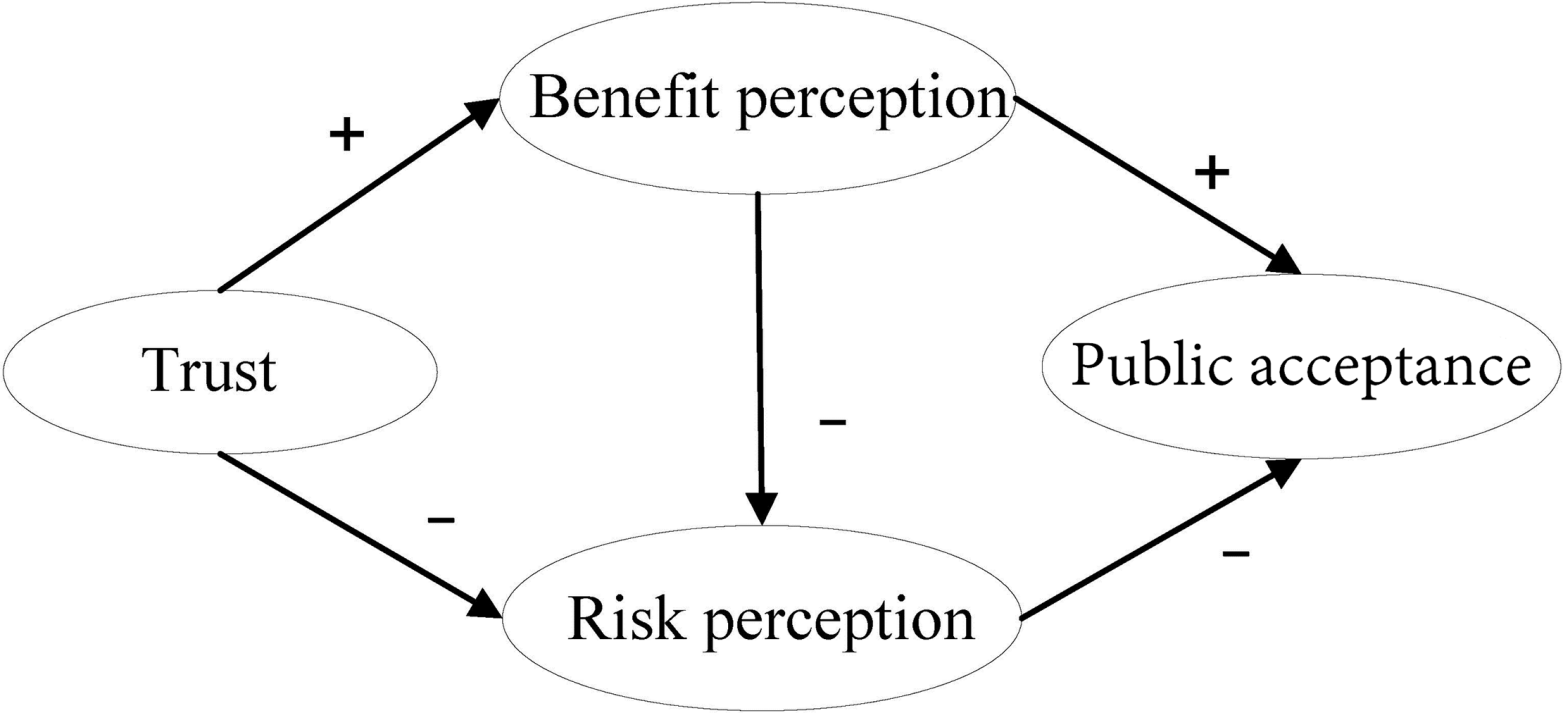
Model of public acceptance of NPPs.

Trust is an important social psychological factor for nuclear energy technology and for nuclear power projects. The research conducted by Visschers et al.[4] on NPP acceptance in Switzerland found that greater public trust could increase benefit perception and decrease risk perception; higher benefit perception could increase public acceptance, while higher risk perception had the opposite effect (see Fig 1). Viklund's research [11], conducted in Western European countries, also showed that there was a negative correlation between public trust and risk perception connected with nuclear technology. Flynn et al. [12] also showed that more trust could offset risk perception of local residents and reduce opposition to a high-radiation waste repository. Venables et al.[32] found that trust and acceptability were positive to predict residents’ attitudes towards the building of a new NPP in the nearby area. If the benefits of the existing NPP were considered outweigh its risks, the residents might have an increase in the acceptability of the NPP at a local site [32]. These studies highlight that trust plays a positive role in the perception of the value of nuclear technology and NPP construction. Hence, previous studies provided consistent findings on the association between trust and development of NPP acceptance among both the public and local residents living with nuclear projects.

However, in some studies trust was defined only as affective or competence but not both. Bella et al. [33] concluded that the crisis over a nuclear waste repository could be attributed to the lack of technological solutions. Thus, when locating the project, trust in the relevant institutions is required; this trust refers only to goodwill trust. Ho et al. [34] proposed that trust could explain the opposition to the planned fourth NPP in Taiwan, because Taiwan's risk management was considered to be inferior to that of Japan; after the Fukushima nuclear accident in 2011, the opponents of nuclear power in Taiwan strongly distrusted their government's ability to manage nuclear emergencies. Obviously, trust here refers to competence trust. Nooteboom [25] highlighted goodwill trust as concerning a partner’s intention to perform according to agreements, while competence trust concerned his/her ability to do so. For goodwill and competence trust, neither can exist effectively without the other.

In the studies mentioned above, trust was measured along a single dimension, which might hide the diverse influences of different kinds of trust. Other variables in trust models may overlap with goodwill trust or competence trust: Visschers et al. [14] explained acceptance of NPPs using variables that included trust, affect, risk perception, benefit perception for climate mitigation, and benefit perception for energy supply. In that study, trust and affect were found to interact strongly, and both trust and affect may influence risk and benefit perceptions. Huijts et al. [35] established a comprehensive framework for energy technology acceptance in which trust directly influenced positive and negative affect, perceived costs, benefits, and risks. Some studies concerning hydrogen acceptance [36,37] provided evidence that trust could influence the inverse relationship between perceived benefit and perceived risk indirectly by moderating negative affect. Goodwill trust itself includes emotional factors, while ‘trust’ and ‘affect’ appear in the same model; it is hard to avoid the overlap of these notions, which might lead to theoretical problems. In another study on public acceptance of recycled water, Ross et al. [38] explained trust with source credibility, fair procedures and group membership identity, where ‘source credibility’ also overlapped with ‘competence trust’.

### Theoretical hypothesis

When people know little about a certain technology, trust in authorities in charge of that technology determines whether or not they will accept the technology. Trust helps individuals use other resources and construct their own ideas [39,40]. Goodwill trust and competence trust describe whether local communities will ignore the risk of a nuclear accident, and whether the residents living with NPPs are confident in NPP authorities (government, experts, and power companies). Those two factors are expected to be key influences on acceptance of NPPs. This paper will explore the influences of trust on the acceptance of NPPs according to the two dimensions above.

The feeling of trust is backed up by empirical evidence, such as credible information concerning the intentions or ability of others [41]. Therefore an incremental process is needed to determine whether an individual, group or organization is trustworthy [42,43]. It seems that goodwill trust in a partner accumulates when that partner has been seen to resolve an issue in an equitable and efficient manner [44]. On the contrary, failures of competence might decrease goodwill trust in cooperation. Johnson & Grayson [17] showed that affective trust (goodwill trust) was supported by cognitive trust (competence trust) in the relationship between consumers and service providers. Heffernan [44] suggested that competence trust had a positive influence on goodwill trust in cross-cultural business-to-business relationships. Thus competence trust might be supposed to correlate positively with goodwill trust in acceptance of NPPs.

**Fig 2:**
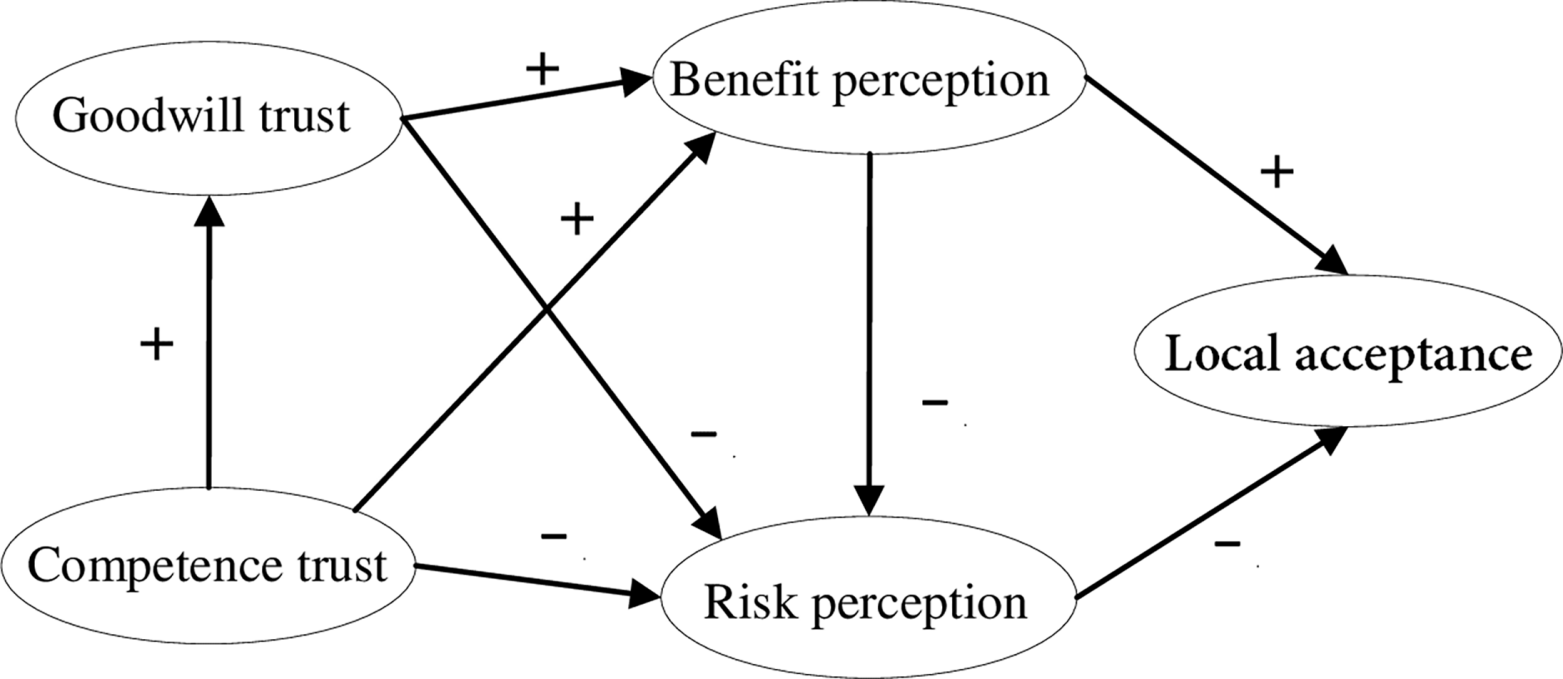
An initial model of local acceptance of NPP with two dimensional trust structure.

Based on explanation model of NPP acceptance developed by Visschers et al. [13] in Switzerland (Fig 1), a new model was established by separating trust into the two latent variables: ‘goodwill trust’ and ‘competence trust’ (Fig 2). In the original model, higher trust was considered to promote NPP acceptance indirectly by increasing benefit perception and decreasing risk perception [13]. Because the past studies reveals one single dimensional trust has significant influences on benefit perception, risk perception, and NPP acceptance, we supposed that two dimensions of trust, goodwill and competence trust might influence NPP acceptance in the same way. Thus we can develop a set of hypotheses as follows.

H_1_. Goodwill trust in NPP authorities will become greater as competence trust increases.

H_2_. Greater goodwill trust in NPP authorities will increase the public’s benefit perception and decrease their risk perception of NPPs.

H_3_. Higher competence trust in NPP authorities will also increase benefit perception and decrease their risk perception of NPPs.

## Data and methods

### Data

A survey on local acceptance of NPPs was carried out in 2014 in Haiyan county, Zhejiang province, China, where the Qinshan NPP was located. There were only 7 NPPs with 18 nuclear reactors in commercial operation during the survey in mainland China [5]. These NPPs were homogeneous in that all of them were coastal projects and held by Chinese government. Among the 7 NPPs, Qinshan NPP was the first nuclear power plant in commercial operation (since 1991), owning the most operating reactors (7 reactors) and surrounded by the denser population. It is distinct the sample is representative for people living with NPPs in China.

Typical attitudes concerning living with the NPPs suggest that the distance of residence or work from the NPP might influence cognitive and affective responses to the NPP. Previous research usually sampled residents within 30 kilometers of an NPP [34,45,46]. Considered that just as the local residents living nearby, the NPPs workers who are working in the sites and living nearby are also facing the risk of NPPs, in the present study, people living or working within a 30 kilometers radius of the Qinshan NPP (including workers at the NPP) were considered as potential respondents. Besides all the workers in the NPP, there were 30 thousand people within 9117 households in Qinshan Town meeting the space standard. With the aid of resident's committees and of Qinshan NPP operator, 400 households and 120 NPP workers were sampled to investigate using stratified random sampling method. All residents were interviewed in their households and the worker were interviewed at sites of NPP. All the respondents invited were above 16 years, and they were asked to fill a questionnaire face-to-face. Overall the survey recruited 504 individuals, 491 of whom gave valid questionnaire responses.

As Table 1 shows, The proportion of male and female respondents was roughly balanced (49.5% were men). Most of the resident respondents were of working age (59.3% for 21–40 years old, 28.1% for 41–60 years old, and 12.6% for others), and had relevant good education (57.6% for college education, 18.3% for senior high school, and 20.6% for or under junior high school), but their annual household income was relatively lower [47.5% under fifty thousand Yuan (RMB), 26.5% between fifty thousand Yuan and one hundred thousand Yuan, and 26.0% above one hundred thousand Yuan] in Zhejiang province, a developed region in China. Thus some local residents have argued that the NPP negatively influenced local economy. While NPP workers had distinctly higher income than the average income of local people. Among the respondents, the staffs of governments and institutions account for 10.8% and other employed workers or self-employed entrepreneurs except the NPP workers (23.8%) account for 49.3%.

**Table 1:** Descriptions of the respondents.

Variables	Categories	Number(Percentage)
Gender	Male	243(49.5%)
	Female	248 (50.5%)
Age	21–40	291(59.3%)
	41–60	138(28.1%)
	others	62(12.6%)
Education	Junior high school and below	101(20.6%)
	Senior high school	90(18.3%)
	College or junior college	283(57.6%)
	Graduate	17(3.5%)
Annual household income	0-50000(RMB)	233(47.5%)
	50001–100000	130(26.5%)
	Above 100000	128(26.0%)
Career	Staff of government and institutions	53(10.8%)
	Other employed workers or self-employed entrepreneurs	242(49.3%)
	Workers at NPP	117(23.8%)
	Retired workers, housewife, students, the unemployed	79(16.1%)

Note: Original data of the variables can be found in S1 File.

### Methods

#### Ethics statement

The study was approved by Ethics Committee of Public Policy and Administration School, Xi’an Jiaotong University. The data was analyzed anonymously and therefore no additional informed consent was required.

#### Setting

We used descriptive statistics and structural equation modeling (SEM) to analyze the level of trust in the NPP and how this trust related to local residents' acceptance of the NPP. Both acceptance of nuclear power as a power-generating mode and nearby construction of the NPP are included. Assessment of questionnaire reliability and validity was carried out in SPSS19.0, and AMOS 17.0 was applied for SEM analysis.

### Measurements of variables

Based on previous related studies and interviews among local residents and workers at the Qinshan NPP, five-point Likert scales adapted to Chinese cultural context were developed to measure all the variables in this study.

Venables et al. [32] measured NPP acceptance by asking subjects if they agreed with the construction of a new NPP locally. Typical adjacency avoidance of an NPP suggests that attitudes to nuclear energy and to construction of a new NPP locally should be different. In the present study a new item was added to measure local residents' acceptance on nuclear energy, namely the respondents were asked whether nuclear energy is acceptable as a form of power generation. In addition, subjects were also asked if they involuntarily to live next to an NPP. These three items measure respondents’ acceptance of NPPs from individual-centered, community-centered and society-centered positions, respectively.

In previous studies, trust has been measured from two perspectives: trust in a partner and trust in the partner's actions. Trust in a partner has been measured by asking respondents whether they trusted the NPP authorities (e.g. [13, 14, 32]). Trust in a partner´s action has been measured by asking how the respondents feel about the risk management plans or activities (e.g. [34]). Given that there is a different mix of politics, media, and industry from those in western societies that may have profound impact on the public and public attitudes towards NPPs, experts rather than the media in China are expected to have more independent influence on the attitude of citizens rather than medias because the latter (be called the mouthpiece of Chinese government) are always consistent with government in their attitudes and behaviors. In the present paper, local residents’ trust was measured by feeling of trust in NPP authorities (including government departments, the operators of NPPs, research institutes and experts).Additionally, all the items for assessment of trust were divided into two dimensions, namely goodwill trust and competence trust. The former focuses on evaluation of sincerity and reliability, while the latter concerns feelings of the capabilities to manage risk.

For benefit perception, previous studies focus mainly on the contribution of NPPs in supplying power [13,14], getting cheaper energy [13,14,47], or regulating climate [14,48]. In China, during power shortage the government might increase electricity price or make resident’s house blackout to reduce electricity consumptions. Moreover, In China, nuclear power plays an important role in reducing environment pollution caused by overuse of fossil fuels, including coal and petroleum, and in supplementing the shortage of electricity caused by rapid economic development. Thus, perception of benefits of NPPs was assessed using items addressing how respondents felt about the effects of nuclear power in reducing local power shortages, in protecting the ecology and environment, and in regulating energy consumption.

Risk perception was evaluated using four measures from the study of Ho et al. [34]: perceived possibility of a nuclear accident, perceived safe distance from an NPP, perceived excess cancer risk for those living within 30 kilometers of an NPP, and perceived possible electricity shortages without NPPs. Actually, a safe distance should be a condition of risk perception, and belief in possible electricity shortages could be attributed to benefit perception [13,14]. Sjöberg & Sjöberg [49] proposed that an important worry about nuclear waste repositories concerned their harm to children and future generations. Hence, risk perception of NPPs was measured with three variables in the present study: perceived health threats, perceived harm to the next generation, and perceived chance of nuclear accidents.

Table 2 shows the primary variables and items used in our study. All scales have good internal consistency. Cronbach’s alpha for the scales of goodwill trust, competence trust, benefit perception, risk perception, and local residents' acceptance are all greater than 0.70. All the standardized factor loadings in the final model are greater than 0.5, indicating that the scales have a good structural validity.

**Table 2:** Variables and measurements.

Variables and measuring items	Standardized factor loadings	Cronbach´s Alpha
**Local acceptance**		0.72
1. I agree with nuclear power as a form of power generation.	0.62	
2. I agree to accept the construction of an NPP locally.	0.79	
3. It is involuntary for me to live next to NPP.	0.64	
**Goodwill trust**		0.73
4. Information about nuclear power provided by the government is objective and reliable.	0.72	
5. Information about nuclear power released by research institutes and experts is objective and reliable.	0.77	
6. The operators of NPPs will deal with nuclear accidents by obeying the government’s order.	0.59	
**Competence trust**		0.78
7. Nuclear power experts can effectively supervise and guide the construction of NPPs, removing dangers.	0.70	
8. The operators of NPPs can effectively control risks involved in NPPs.	0.78	
9. The risk of nuclear energy can be precisely evaluated by current sciences and technologies.	0.74	
**Benefit perception**		0.79
10. Nuclear power is clean energy, the use of which is helpful for environmental protection.	0.74	
11. Our country should produce more nuclear power, and gradually change the structure of energy consumption.	0.73	
12. I will agree to have a local NPP if there is an electricity shortage where I live.	0.76	
**Risk perception**		0.76
13. An NPP might harm me and my health.	0.69	
14. The risk of NPP is devastating.	0.68	
15. The construction of an NPP is a potential danger for my descendants.	0.78	

Note: Original data of the variables can be found in S1 File.

## Results

### Trust and acceptance by local residents

**Fig 3:**
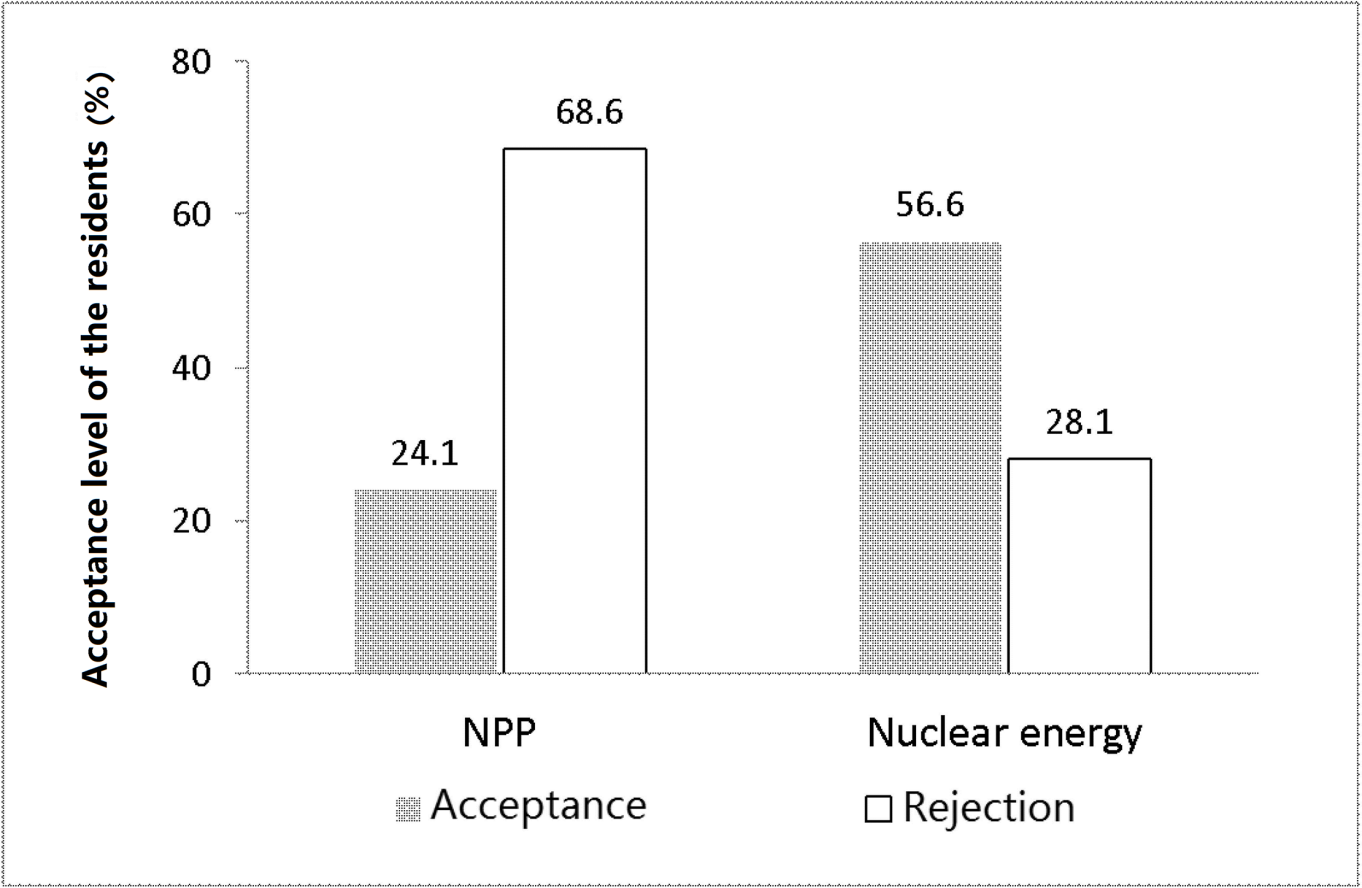
Comparison of local acceptance of nuclear energy and NPPs.

Fig 3 shows that the respondents supporting (i.e., who selected ‘strongly agree’ and ‘agree’) and opposing (i.e., selected ‘strongly disagree’ and ‘disagree’) the construction of the NPP locally account for 24.1% and 68.6%, respectively. Obviously the number of people against the construction of NPP locally is greater. However, the respondents supporting and opposing the usage of nuclear power account for 56.6% and 28.1%, respectively, so people with a positive attitude to the usage of unclear power are in a substantial majority. The different attitudes towards NPP construction and nuclear energy suggest that the residents in China is typically adjacency avoidant, ‘we accept and welcome the usage of nuclear energy, but please do not build an NPP in my back yard’.

**Fig 4:**
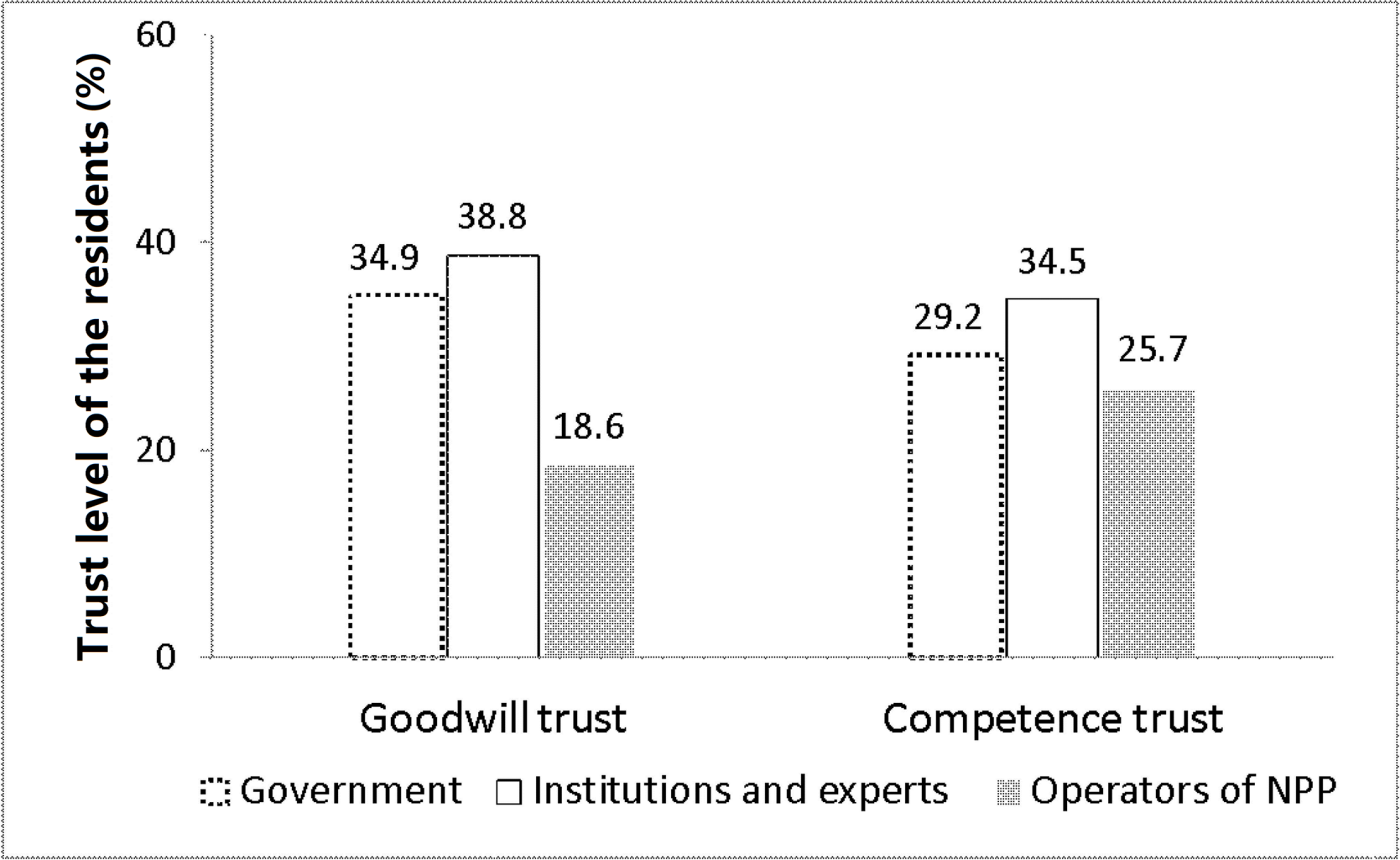
Goodwill trust and competence trust of local residents in NPP authorities.

Fig 4 shows results on goodwill trust. More than one third of the respondents trust in government, research institutions, and experts, but only 18.6% of respondents trust the operators of NPPs. Obviously the government, research institutions, and experts are thought to be more reliable than the operators of NPPs. As to competence trust, the percentages of respondents who trust government, research institutions and experts, and the operators of NPPs are 29.2%, 34.5%, and 25.7%, respectively; these are not significantly different. These findings indicate that NPP authorities in China, especially the operators of the NPP, are less likely to be trusted.

### How trust affects NPP acceptance

The influence of trust on NPP acceptance is explored using maximum likelihood (ML) estimation in the initial model (Fig 2). Root mean square error of approximation (RMSEA) is widely used to evaluate goodness-of-fit of structural equation models, whose value >.05 and < .08 (RMSEA = .055) indicates reasonable fit rather than good fit [50].We also use the normalized chi square (X^2^/df) to evaluate model fit, with values (X^2^/df = 2.457) between 1.00 and 3.00 indicative of acceptable fit [51]. As recommended by Hu & Bentler [52], a cutoff value >.95 for the Tucker-Lewis Index (TLI), Bollen's (1989) Fit Index (BL89, or NFI), and Comparative Fit Index (CFI); and a cutoff value < .08 for standardized root mean squared residual (SRMR) also suggest that the initial model is acceptable but not ideal (BL89 = .938, CFI = .962, TLI = .951, PNFI = .732, SRMR = .035).

Modification indices (MIs) indicate that the model’s fit can be improved by allowing a correlation between the error terms of the items. The MIs of items 6 and 8 were higher than that of the others. Item 6 assessed the belief in law-abiding treatment of accidents by the operators of NPPS, and item 8 assessed the belief in risk control abilities of these operators. Some factors that improve the belief in law-abiding behavior by the operators of NPPs, may also improve the belief in risk control abilities of these operators. Therefore, we covaried error term of item 6 with that of item 8.

The critical ratio (C. R.) of the paths in the initial model shown in Table 3 indicate that the four paths of ‘Goodwill trust→Benefit perception’, ‘Goodwill trust→ Risk perception’, ‘Competence trust→Benefit perception’ and ‘Competence trust → Risk perception’ are not significant.

**Table 3:** Path parameters in the initial model.

Paths	Estimate path coefficients	Standardized Error	Critical Ratio	P
Goodwill trust→ Benefit perception	-0.21	0.96	-0.22	0.83
Goodwill trust→ Risk perception	1.92	2.00	0.96	0.34
Competence trust→ Goodwill trust	0.99	0.07	14.36	***
Competence trust → Benefit perception	0.92	0.98	0.94	0.35
Competence trust → Risk perception	-2.27	2.09	-1.09	0.28
Benefit perception → Risk perception	-0.34	0.12	-2.75	**
Benefit perception → Local acceptance	0.67	0.07	9.96	***
Risk perception → Local acceptance	-0.61	0.08	-8.21	***

Note

** significant at P < .01

***significant at P < .001.

**Fig 5:**
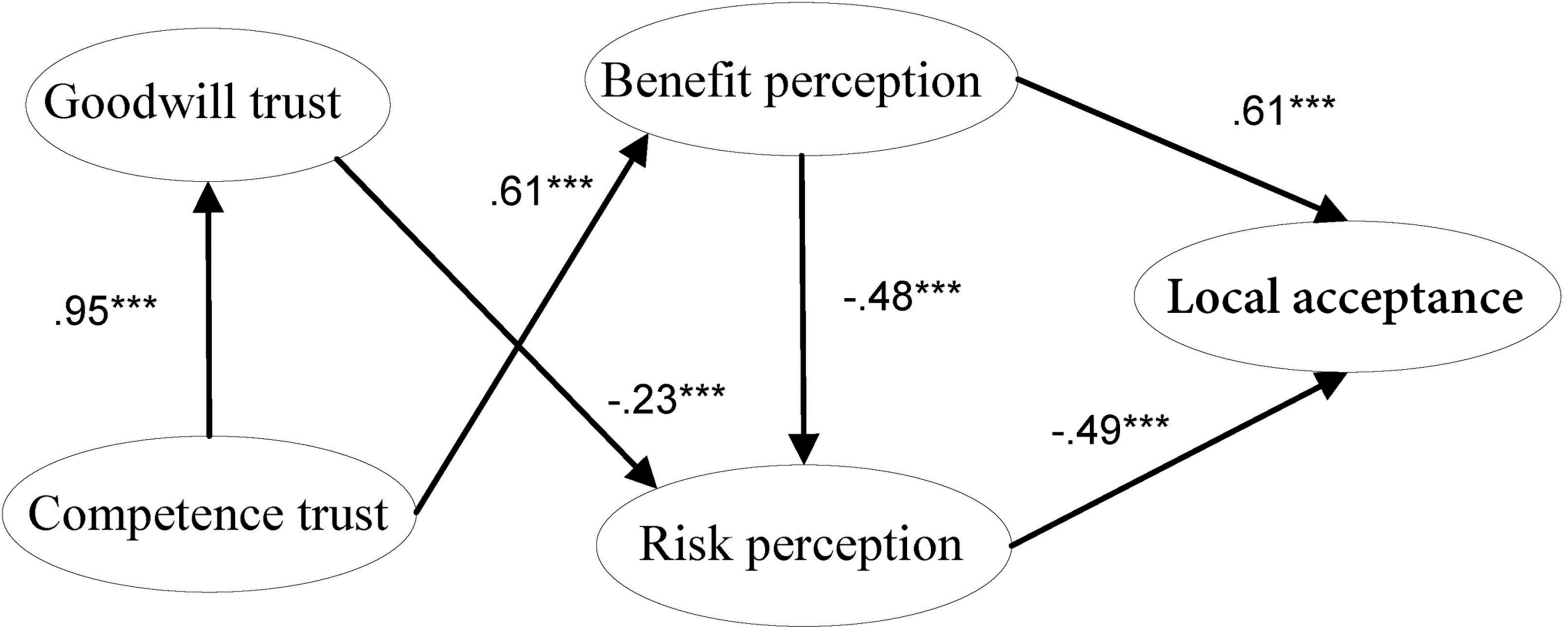
Modified trust model for local acceptance of NPPs. Note: * significant at P < .05, ** significant at P < .01, ***significant at P < .001.

Visschers et al. [13] and Huijts et al. [35] have shown that trust determines some part of the perceived benefit and risk of NPP. In the initial model (Fig 2), the correlation between trust and benefit perception, and the correlation between trust and risk perception are not confirmed, which is inconsistent with previous findings. This inconsistency may suggest that the causal paths of goodwill and competence trust to benefit and risk perception are different. Hence, we modify the initial model by trimming and removing both paths from ‘Goodwill trust→ Benefit perception’ and ‘Competence trust → Risk perception’ that have insignificant C.R. and high P values (>.05). The final model is shown in Fig 5.

Goodness of fit of this final model is improved (X^2^/df = 2.058, BL89 = .947, CFI = .972, TLI = .964, PNFI = .738, SRMR = .035, RMSEA = .046). In addition, all the paths in the final model are significant at the 0.001 level (as Fig 5 shows), and the standardized factor loadings of each item are greater than 0.50 (shown in Table 2). Clearly the final model is more acceptable.

In our modified model (Fig 5), both benefit perception and risk perception are significantly correlated with local residents' acceptance, but the impact of benefit perception is positive, and the impact of risk perception is negative. Benefit perception and risk perception are negatively correlated, while goodwill trust and competence trust are positively correlated. The negative correlation between goodwill trust and risk perception of NPPs is statistically significant, while competence trust is positively and significantly correlated with benefit perception of NPPs. In addition, it is found that the correlation between goodwill trust and benefit perception, and the correlation between competence trust and risk perception are not statistically significant.

## Discussion

### Trust of NPPs in China

In consistent with the findings of Globescan investigation in that there is a global bias against nuclear power technology as measured by public acceptance and trust in nuclear power, our study confirmed that this distrust in NPPs also exists in China. As Globescan investigation revealed that over 60% general Chinese (not living around NPP sites) accepted nuclear power [7], however, our survey shows that only 24.1% local residents accept the construction of NPPs nearby. The degree of trust in government departments, research institutions, experts, and the operators of NPPs is also far less than half. Obviously, the plans to expand use of nuclear power in China are being carried out without much local communities’ trust. For nuclear energy to be successful in China, authorities in charge of NPPs need to increase public trust especially residents’ trust in nuclear power.

### Trust mechanism of local residents near NPP

In previous studies, trust in nuclear power (or NPPs) was considered as a one-dimensional concept. Here we divided it into two dimensions, goodwill trust and competence trust, and explored their relationship with acceptance of NPPs. Our results reinforce and extend those found in the study of Visschers et al. [13], including that greater benefit perception and less risk perception should improve NPP acceptance directly. Greater benefit perception can indirectly improve NPP acceptance by decreasing risk perception. We also found that greater goodwill trust in NPP authorities might decrease risk perception of NPPs, and greater competence trust can increase benefit perception. Thus, NPP acceptance can be promoted by goodwill trust and competence trust indirectly.

Our study also extends the current understanding of local residents’ trust in NPPs. As to the two dimensions of trust, greater goodwill trust has a negative impact on risk perception. This may be attributed to NPPs being a facility with high technical barriers and high local risk avoidance. The residents are unable to participate in its risk management and they must depend entirely on NPP authorities for security assurance. As a result, if NPP authorities try their best to show sincerity, credibility and hope, risk perception of local residents caused by NPPs might be partly relieved. Moreover, we also found that greater competence trust has a positive impact on benefit perception. It may be that greater competence trust in NPP authorities reflects people’s greater confidence in the capabilities of NPP authorities and in NPPs security management, which are necessary for the belief that the operation of nuclear reactors is safe and that perceived benefits can be protected from nuclear accidents. For people next to NPPs the key point to increase benefit perception, is given good feelings of safe nuclear power technologies and perfect NPPs management.

Unexpectedly, both the impact of competence trust on risk perception and the impact of goodwill trust on benefit perception are not significant, which may be partly explained by the findings of Vikund [11], who compared the relationship between public trust and perceived nuclear risks within four European countries, and also concluded that general trust (similar to goodwill trust) can explain perceived risk better than specific trust (similar to competence trust). Das & Teng [8] provided another possible explanation in the field of business trust. They proposed that a firm’s goodwill trust in a partner would reduce the perceived risk in forming an alliance (perceived relational risk), but not the perceived performance risk, while competence trust in the partner would reduce perceived performance risk from an alliance, but not its perceived relational risk [8]. In our study, the residents’ goodwill trust in NPP authorities might also reduce the perceived relational risk between local communities and NPP authorities, but not the risk from the perceived performance of NPPs (increasing benefit perception). On the contrary, competence trust means positive expectation from others in the risk context [8], and because individuals are unable to deal with the risk by themselves, they have to depend strongly on partners with knowledge, resources, and ability [8], who might be believed to be associated with better performance of NPPs.

### Limitations

There are several limitations to our study. We considered people living or working within a 30 kilometers radius of the NPP as sample population, but the distinction was not clear between employees in the NPP and people not connected to the NPP business. The extent to which our findings can be generalized to other NPP even to the whole country needs to be addressed by replicating the study in other NPPs or with a representative nationwide sample. In addition, it has been ignored that residents living in different distance from the NPP might have different attitudes to the NPP. Another limitation is that benefit perception of NPP is measured with the items looking like the feelings of public benefits. Previous studies also addressed benefit perception of NPP in that way (e.g. [13–14,47–48]). In fact the feelings of personal benefits are the measuring target, which is involved in people’s.

## Supporting information

S1 File Supplementary data for Tables 1 and 2. (XLS)
